# Investigation of chimeric reads using the MinION

**DOI:** 10.12688/f1000research.11547.2

**Published:** 2017-08-16

**Authors:** Ruby White, Christophe Pellefigues, Franca Ronchese, Olivier Lamiable, David Eccles

**Affiliations:** 1 Malaghan Institute of Medical Research, Wellington, 6242, New Zealand

**Keywords:** chimerism, MinION, interferon, amplicon, R9.4, signal, nanopore

## Abstract

Following a nanopore sequencing run of PCR products of three amplicons less than 1kb, an abundance of reads failed quality control due to template/complement mismatch. A BLAST search demonstrated that some of the failed reads mapped to two different genes -- an unexpected observation, given that PCR was carried out separately for each amplicon. A further investigation was carried out specifically to search for chimeric reads, using separate barcodes for each amplicon and trying two different ligation methods prior to sample loading. Despite the separation of ligation products, chimeric reads formed from different amplicons were still observed in the base-called sequence. The long-read nature of nanopore sequencing presents an effective tool for the discovery and filtering of chimeric reads. We have found that at least 1.7% of reads prepared using the Nanopore LSK002 2D Ligation Kit include post-amplification chimeric elements. This finding has potential implications for other amplicon sequencing technologies, as the process is unlikely to be specific to the sample preparation used for nanopore sequencing.

## Introduction

High-throughput DNA sequencing is a rapidly evolving field with new methods and applications introduced almost weekly ^1^. One of the most recent sequencing technologies available on the market is the MinION sequencing device from Oxford Nanopore Technologies (ONT) ^2^. A brief overview of MinION sequencing technology is discussed in our previous study on mitochondrial genome assembly ^3^.

Instead of exploiting base-pairing as in the sequencing-by-synthesis approach used by Illumina and others, nanopore sequencing uses an electronic sensor to detect DNA via a change in electric current (reviewed in 4). The MinION’s flow cell is comprised of 2048 wells containing a membrane perforated by nanopores. Ligated with a molecular motor, a single stranded DNA molecule passes through the pore, altering the recorded current. After the electronic sequencing is carried out, a software base-calling algorithm transforms the current trace into a modelled DNA sequence. The advantages of the MinION are rapid library preparation, portability ^5, 6^, long molecule sequencing ^7^, and sequencing of non-model modifications of the DNA strand ^8^. Recent improvements in the chemistry of the MinION have overcome the majority of issues associated with low yield and high error rates that have limited the range of its application. The MinION sequencing device has now been successfully used to sequence genomes of a wide range of sizes, from bacterial and viral genomes ^9, 10^, amplicon sequencing such as bacterial 16S rRNA sequencing ^11^, and more recently a human genome ^12^. The MinION has also been used for cDNA sequencing ^13^, for detecting DNA methylation patterns without chemical treatment ^8, 14^, and for direct RNA sequencing with detection of modified 16S rRNA nucleotides ^15^.

Using R9.4 flow cells we have evaluated the MinION technology for the amplicon sequencing of highly similar genes. Since we have an interest in the interferon response during helminth infection ^16^, we sequenced the type I interferon (IFN) family. Type I IFNs are a family of intronless antiviral response genes comprised, in mice, of 14 highly homologous *Ifna* members, as well as the genes *Ifnb*, *Ifnk* and *Ifne* ^17^. In humans, sequence similarity across the 14 members of the *Ifna* genes is 70–80%, with a further 35% sequence similarity between *Ifna* and *Ifnb*. Type I IFN has both an important role in innate antiviral immunity and in mounting adaptive T helper cell responses ^16, 18^. Building on previous observations, we aimed to identify which type I IFN member(s) were responsible for driving the type I IFN signalling in our infection model.

Due to the high homology between the *Ifna* family genes, accurately detecting quantitative expression of the different gene members by Sanger sequencing or next generation sequencing is difficult. We instead employed nanopore sequencing, which allowed us to acquire full-length reads from each individual sequence that were amplified by the PCR reaction. We aimed to determine the relative quantities of the various *Ifna* family and *Ifnb* transcripts, in helminth-treated mouse ear tissue using the MinION; therefore enabling both the differentiation between the various *Ifna* genes, and the potential to perform quantitative analysis.

## Methods

*Nippostrongylus brasiliensis* was originally sourced from Lindsey Dent of the University of Adelaide, South Australia and has been maintained for 22 years by serial passage at the Malaghan Institute. Female Lewis rats were bred and used for maintenance of the *N. brasiliensis* life cycle when 4 months of age (and weight over 150g), as outlined in Camberis *et al.* ^19^.

Two 8-week-old C57BL6/J male mice (Jackson Laboratories, approx 23g), housed and bred at the MIMR under specific pathogen free conditions respecting the local and New Zealand ethic guidelines, were chosen for the investigation. 300 dead infective *N. brasiliensis* L3 larvae were injected intradermally in each ear of one mouse in 30uL PBS after anaesthesia with an intraperitoneal injection of 200uL ketamine/xylazine. The other mouse was similarly euthanised and injected intradermally in each ear with 30uL PBS. The mice were euthanised in a *CO*_2_ chamber 3h post injection and ears (approx 27–30mg in weight) were immediately harvested and conserved in RNALater at 4C for <1h. RNA extraction of each whole ear was done in 1mL of Trizol following the products’ guidelines (ThermoFisher). cDNA was synthesised using the High Capacity RNA-to-cDNA kit (Applied Biosystems), according to the manufacturer’s instructions. Only the cDNA from the *N. brasiliensis*-treated mouse was used for this investigation.

**Figure 1.**
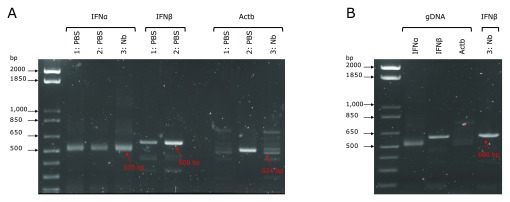
Gel-electrophoresis image of PCR products amplified for this investigation. ( **A**) Amplicons were observed for Ifna and Actb from both PBS treated (1&2, not sequenced in this investigation) and *Nb*-treated (3) samples at the expected sizes of 535 bp, and 524 bp respectively. The Ifnb gene from the *Nb*-treated sample (3) failed to amplify during this first attempt. ( **B**) A repeat amplification of Ifnb from *Nb*-treated sample was carried out, producing a single band of approximately 600bp. This was run alongside amplicons of Ifna, Ifnb and Actb from genomic DNA; however genomic amplicons were not used for subsequent MinION sequencing.

*Ifna*, *Ifnb*, and *Actb* amplicons were generated using specific primers: *IfnaF* (ATGGCTAGRCTCTGTGCTTTCCT) and *IfnaR* (AGGGCTCTCCAGAYTTCTGCTCTG) ^20^; *IfnbF* (CTGGCTTCCATCATGAACAA) and *IfnbR* (GCAACCACCACTCATTCTGA); and *ActbF* (AGGGAAATCGTGCGTGACAT) and *ActbR* (ACGCAGCTCAGTAACAGTCC). PCR amplification was performed using Phusion High-Fidelity PCR Kit (Thermo Scientific), see Figure 1. PCR products were cleaned using QIAquick PCR Purification Kit (QIA-GEN) and verified by gel electrophoresis.

*Ifna* cDNA were amplified by PCR using primers designed across a highly-conserved region of all *Ifna* coding sequences, which resulted in a mixed PCR product containing all 14 *Ifna* genes. cDNAs of *Ifnb* and *Actb* were amplified separately and used as quantification controls. Altogether, the three pooled amplicons were loaded into a flow cell and sequenced. Among the reads that we obtained, we noticed long chimeric reads comprising of two or more sequences from different amplicons. We decided to further examine this phenomenon.

Ethics approval for maintenance of the *N. brasiliensis* life cycle is overseen and approved by the Victoria University of Wellington Animal Ethics Committee. C57BL/6J mice were originally obtained from The Jackson Laboratory, Bar Harbour, Maine, USA, and maintained at the Biomedical Researc Unit of the Malaghan Institute of Medical Research by brother X sister mating. Breeding pairs were refreshed regularly to maintain the genetic integrity of the strain. Mice were maintained in specific pathogen-free conditions and all mouse experiments were approved by the Victoria University Animal Ethics Committee (permit number 23907) and carried out according to institutional guidelines.

### Library preparation

**Figure 2.**
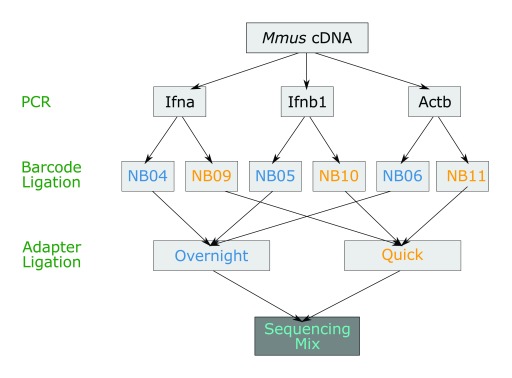
Sample preparation workflow demonstrating the steps used to aid in the identification of the stage at which chimeric reads were formed. Mouse cDNA was extracted and separately amplified for three different amplicons. The amplified product was then separated and barcoded based on the intended ligation process. Barcoded products were pooled and ligated to adapters via the overnight or the quick ligation method, then finally pooled together for sequencing.

The ONT Native Barcoding Kit (EXP-NBD002) and 2D Ligation Sequencing Kit (SQK-LSK208) were used to prepare the samples for sequencing, as per the manufacturer’s protocol. Briefly, purified PCR amplicon products were blunt-ended, ligated with barcode sequences, pooled in approximately equimolar amounts, then ligated with flow cell adapters and a hairpin linker. In order to explore the effect of ligation method on the degree of chimerism, two different adapter/hairpin ligation reactions were carried out: one using the standard quick (10-minute) ligation, and the other using an overnight ligation at 4° Celsius. No additional adapter-free controls were used; it has been our prior experience that sequencing does not proceed in a callable fashion unless adapter sequences are present. The barcoding scheme used in the library preparation is shown in Figure 2. Samples were quantified after barcoding for overnight ligation (2.14 *ng/µl*, 2.54 *ng/µl* and 2.56 *ng/µl* for *Ifna*, *Ifnb*, and *Actb* respectively) and for quick ligation (2.13 *ng/µl*, 2.68 *ng/µl* and 2.45 *ng/µl* for *Ifna*, *Ifnb*, and *Actb* respectively). These samples were normalised and pooled together to give 26.6ng each in 33.1 *µl* distilled water for ligation. After adapter ligation, the quick ligation method showed no detectable nucleic acid, as seen using a fluorescence quantitation with the Quantus fluorometer (Promega), while the overnight ligation quantified at 0.239ng/ *µl*. We decided to pool the samples together anyway, and were pleasantly surprised to discover a substantial proportion of reads from quick-barcoded sequences.

### Base-calling

Reads were initially base-called during the sequencing runs in January 2017 using Metrichor 2D basecalling, from MinKNOW v1.3.25. An initial analysis of called reads demonstrated substantial disagreement between base-calls and the raw signal (e.g. hairpin adapter sequences matching multiple times when the signal showed only one present), so reads were recalled as in March 2017 using Albacore v0.7.5.

## Results and discussion

**Figure 3.**
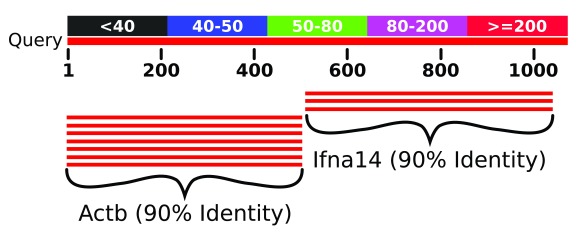
A chimeric read that was discovered during the preliminary investigation of interferon expression. This read mapped to both beta-actin and interferon alpha, suggesting that a ligation of sequence had occurred, either during sample preparation or *in-silico*.

During the initial MinION sequencing run to investigate the expression of *Ifna*-family members in mice (comparing with *Ifnb* and *Actb* transcripts), we encountered issues with 2D base-calling through the Metrichor web service, which seemed to be due to failed alignment of component 1D strands. A BLAST search on some of the longest base-called 1D reads led to a discovery that some reads had multiple mappings to our target *Ifna*-family members. Further exploration of the data demonstrated a situation in which both *Ifna* and *Actb* sequences were present in the same read (see Figure 3). This was an unexpected result; we had carried out separate PCR reactions for each transcript, so were not expecting reads to appear that mapped to different transcripts. Our conclusion was that chimeric ligation of input DNA was occurring at some stage during the sample preparation process, but all we were able to determine at the time was that this chimerism was happening some time after the PCR, but before the sequencing. The present experiment was designed in light of these prior results to more easily quantify the degree of ligation that was happening.

### Read counts

Despite using a 2D ligation chemistry in the sample preparation, and selecting out hairpin-containing reads using streptavidin beads, the majority of reads could not be called as an aligned 2D sequence: of 329,591 sequenced reads, 299,124 were base-called by Albacore, and 1005 (0.3%) of these base-called reads had an aligned 2D sequence (see Supplementary File 1). Any called reads that were not called as 2D were processed further as 1D sequence, i.e. the remaining 298,119 (99.7%) of called reads.

Discussions with ONT staff, in particular Forrest Brennen, during the London Calling conference in 2017 provided insight into what had caused the failure in 2D base-calls. Oxford Nanopore Technologies introduced a chemistry upgrade for their 2D ligation sequencing kits that produced a different, and more obvious, hairpin signal with three peaks rather than two. This modified hairpin signal was the one that the Metrichor and Albacore base-callers were looking for in January 2017 and March 2017 respectively. However, the 2D barcoding kit that we used still had the old hairpin adapter included, and this meant that the base-callers ignored the hairpin region and attempted to call the entire sequence as a 1D read. Oxford Nanopore Technologies subsequently updated their Albacore base-caller to correct this error for 2D barcoded reads, but due to discontinuing the 2D chemistry in preference to the faster and more accurate 1D ^2^ chemistry, the 2D base-caller is no longer developed or included in Albacore. We were able to obtain from ONT the latest, and only, Albacore version that included this fix (version 1.2.4), and recalling reads showed substantial improvement in detecting 2D sequence: 40.8% of reads were called as 2D reads, which was much closer to the 48.6% of reads that we found with a detectable hairpin adapter in the 1D base-called sequence.

### Read mapping

Called 1D reads were mapped to *Actb*, *Ifnb1*, an *Ifna* consensus sequence, additional interferon sequences, the ONT control strand sequence, and known ONT adapter sequences (see Supplementary File 2) using LAST v833 ^21^. A total of 261,183 reads (87.6% of called 1D reads) were discovered that mapped to at least one known amplicon and/or barcode sequence.

### Categorisation of chimeric reads

**Figure 4.**
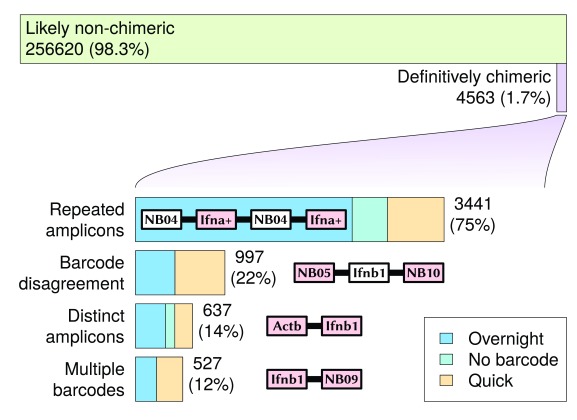
Definitively chimeric reads mapped during the sequencing run. Chimeric read categories are not disjointed: different categories may intersect with each other. Reads that mapped to repeated identical, but reverse-complemented sequences, are not included in these chimeric results, as it was not possible to distinguish at the base sequence level between such a duplicated sequence fragment and a 2D read with hairpin.

Using a process of elimination, a total of 4563 reads (1.7% of amplicon or barcode-mappable 1D reads) were discovered with base-called sequences that were definitively chimeric (see Supplementary File 5). These reads mapped at least once to either one of the three amplicon sequences, or at least once to one of the six barcode sequences. These were broken into four categories (with some overlap) based on the observed combinations of barcode and amplicon sequences (see Figure 4):

1. Repeated identical amplicons aligned in the same direction
2. At least two distinct amplicons
3. At least two distinct barcode
4. Disagreement between barcode and amplicon

A more complete count of different categories of chimerism (for those observed at least five times) can be found in Table 1. The highest proportion of chimeric reads were associated with repeated identical amplicons, with 3441 reads seen (75% of all definitively chimeric reads). This suggests that an amplicon sequencing procedure will be particularly susceptible to read chimerism, as the same sequence will appear in increased abundance compared to an untargeted sequencing approach. One potential mechanism for this is that the identical sequences encourage the formation of complex base-pairing structures (e.g. quadruplexes) that bring the ends of similar sequences closer to each other. The low-temperature overnight ligation resulted in a much higher proportion of repeated amplicons than the quick ligation; in this case it appears that the quick ligation was better at reducing the occurrence of chimeric reads, despite prior expectations.

**Table 1.** Chimeric read counts split into categories depending on the number of amplicons and barcodes seen. Only categories with a count of 5 or more are displayed.

Amplicon count			Barcodes seen			Read count
Ifna	Ifnb1	Actb	overnight	quick	disagreement	
0	0	2	○			876
2	0	0	○			803
0	2	0	○			704
2	0	0		○		378
1	0	0		○	○	246
0	0	2		○		224
2	0	0				201
1	0	1	○		○	140
0	2	0				125
1	0	1		○	○	108
1	1	0	○		○	99
0	1	1	○		○	79
0	0	2				67
0	1	0		○	○	64
1	1	0		○	○	48
0	0	1	○		○	42
1	1	0				41
0	0	1		○	○	37
1	0	1				36
0	1	0	○		○	34
0	1	1		○	○	31
1	0	0	○		○	27
0	1	1				25
0	0	0		○		19
3	0	0	○			15
0	2	0		○		15
2	0	0		○	○	7
0	0	3	○			7
0	3	0	○			6
0	0	0	○			5

Of the definitively chimeric reads, 2869 included at least one overnight barcode (1.8% of 159,188 amplicon-mapped reads with an overnight barcode), and 1203 included at least one quick barcode (2.6% of 45,850 amplicon-mapped reads with a quick barcode). While it appears that the use of overnight ligation has helped somewhat to reduce chimeric reads, a substantial proportion of chimeric reads still remain.

If a cassette of adjacent *Ifna* genes were transcribed together, it is possible that this cassette could be amplified together as a single sequence. These sequences would appear to be chimeric (and fall into the "Repeated amplicons" category), but would not have any intermediate barcodes. The count similarities for repeated *Ifna*, *Ifnb1* and *Actb* genes suggest that this cassette amplification is not happening at any significant level.

### Categorisation of non-chimeric reads

**Figure 5.**
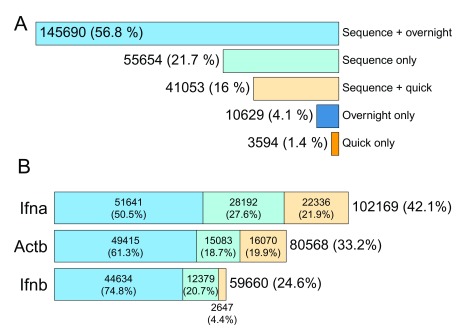
Amplicons mapped from basecalled non-chimeric reads. ( **A**) Amplicon counts split by barcode type. ( **B**) Sequence only, quick barcode, and overnight barcode counts for amplicon-mapped sequences.

After elimination of definitively chimeric reads, 256,620 reads remained that appeared to map uniquely to single sequences (see Figure 5). A small proportion of these sequences (14,223; or 5.5%) had detectable barcode sequences, but did not map to any amplicons (i.e. mappable to an overnight or quick barcode sequence only). It is expected that these unmapped barcoded sequences were unamplified mouse cDNA sequences.

A difference in read counts was observed between overnight-barcoded sequences and quick-barcoded sequences (77.8% overnight, 22.2% quick), which was consistent with the difference in input amount observed during sample preparation. An attempt was made during sample preparation to add in the three different amplicon preparations in equimolar quantities, which was more successful for the *Actb* preparation (33.6%) than it was for the *Ifna* and *Ifnb* preparations (42.7% and 23.7%, respectively).

An additional categorisation of *Ifna* family members (see Supplementary File 3) was attempted, but is not presented here as it detracts from the main chimeric read investigation. Intermediate results and a processing script from this categorisation are available in verbose form as Supplementary File 4.

### Read signal confirmation of chimerism

**Figure 6.**
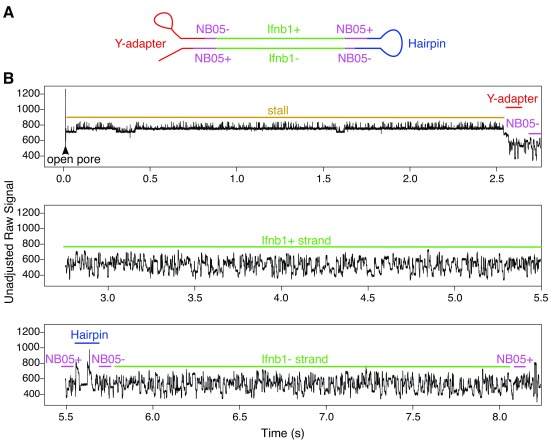
Demonstrative raw signal for a non-chimeric read (from an *Ifnb1* amplicon). The recorded signal for this read starts with a very long period of 7s in the open pore state, followed by a short stall of 0.3s, then a coding *Ifnb1* sequence that took 2.5s to transition through the pore, then an NB05-flanked non-coding *Ifnb1* sequence that took 2s seconds to transition through the pore. *Note: These figures have been annotated with approximate region boundaries based on the order of hits to the base-called sequence.*

A few of the reads were investigated at the raw signal level to make sure that the electrical trace was in agreement with the base-called signal. A demonstrative signal trace for a non-chimeric 2D read comprising of a single barcode-adapted amplicon is shown in Figure 6. Read traces typically began with a high-current (but relatively uniform) open pore state, followed by an intermediate stall signal (also fairly uniform), after which the highly variable sequence trace begins. Hairpin adapters could be easily identified in the raw signal as a bridge structure a little over halfway through a 2D sequence.

**Figure 7.**
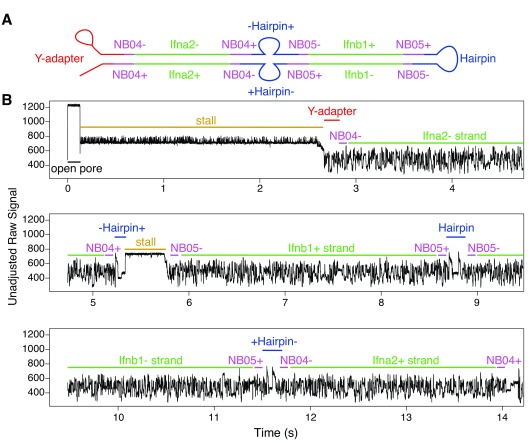
Demonstrative raw signal for a chimeric read (containing a strand dissociation event and two separate hairpin events within the same base-called sequence). The recorded signal begins with a very short open pore state (0.1s), followed by a long stall (2.5s), then an NB04-flanked *Ifna2* non-coding sequence with a transition time of 2.5s. At this stage there appears to be the beginning of a hairpin sequence that is finished by a pore stall. This was followed by a coding *Ifnb1* sequence with a transition time of 2s, then a hairpin, then an NB05-flanked non-coding *Ifnb1* sequence (2.5s), and finally an NB04-flanked coding *Ifna2* sequence (2.5s). Barcodes detected from this read (NB04/NB05) suggest that the chimeric sequence was likely formed during overnight ligation.

A number of situations were observed in the base-called sequence where ligation during sample prep seems to have occurred, and in some cases this ligation resulted in multiple hairpin adapters being ligated in the same sequence. One such occurrence of this is seen in Figure 7, where two barcoded overnight sequences from two different amplicons ( *Ifnb1* and *Ifna2*) were joined together. Because two amplicons were concatenated, this ligation must have happened after the barcoding step of sample preparation (i.e. during adapter ligation).

This finding has potential implications for other sequencing technologies, as the ligation process used for sample preparation is unlikely to be specific for nanopore sequencing. The formation of chimeric reads during sample preparation may be one explanation for the index switching phenomenon seen in Illumina-sequenced reads (e.g. see 22– 24), and presents a substantial problem for dual-indexed reads where identical indexes are used for different samples. Where dual-indexed reads are not used, ligation of reads with the same index may still be problematic depending on the particular sequencing application.

### *In-silico* chimerism (1D ^2^)

**Figure 8.**
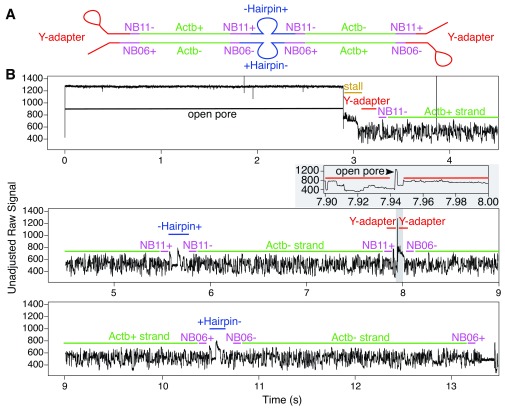
Demonstrative raw signal for a read (base-called as chimeric) that appeared to be from two different ligation preparations. The recorded signal begins with a long open pore period (2.9s), and a short stall (0.1s), followed by NB11-flanked coding and non-coding *Actb* sequences (transition time of 2.5s for each). There is a very short open-pore blip at around 8s, followed by a short stall (0.1s), then NB06-flanked coding and non-coding *Actb* sequences (transition time of 2.5s for each).

There were 8 instances where both an overnight and a quick barcode were observed in the base-called sequence. In all such cases, there appears to have been a very short pore-protein dissociation between the sequencing of the two sequences (i.e. these were chimeric reads formed from *in-silico* ligation). The dissociation was only noticeable after inspecting the raw signal: a very short blip in the signal that matched the open pore current (e.g. see Figure 8).

It is likely to be the case that similar situations involving fast pore reloading are present in other reads, but not easily detectable from the called sequence because other barcode/amplicon combinations fit the expected base calling pattern. Considering that this situation can happen with non-identical sequences, software that is able to flag the presence of dissociation and/or stall events that are not at the start of the raw signal would be useful, as these features suggest that the base call is not likely to be a correct single sequence.

The release of ONT’s R9.5 flow cells and 1D ^2^ base-calling exploits this phenomenon of fast sequence loading into pores in order to produce high-accuracy reads derived from a combined template/complement base-call (i.e. replacing the current hairpin-based 2D call). This replaces the 2D sample preparation process that we used for this investigation (see 25).

## Conclusions

It is apparent from our investigation that chimeric reads can exist in the output of sequencing runs, and we recommend that researchers consider this possibility when interpreting their own results. As a result, it is advisable to include easily-detectable adapters when sequencing DNA. These adapters, particularly if present at both ends of a sequence, will help substantially in the identification (and if necessary, filtering) of concatenated sequences that are not native to the sample.

Although a non-negligible 1.7% of reads were found to have post-amplification chimeric elements, careful quality control of reads after long-read sequencing should be able to identify and exclude the majority of chimeric reads that are produced during a sequencing run.

## Data availability

The data referenced by this article are under copyright with the following copyright statement: Copyright: © 2017 White R et al.

Raw read signal and basecalled reads have been uploaded to ENA under accession number PRJEB20601. Additional supplementary scripts used for FASTQ file filtering, mapping, and raw signal investigation are available as part of David Eccles’ bioinformatics script repository (doi, 10.5281/zenodo.556966) ^26^. The following scripts from that repository were used for intermediate discovery and result generation:

**maf_bcsplit.pl** Converting MAF format to machine-readable CSV with forward-oriented location information

**pos_aggregate.pl** Merging adjacent MAF matches to the same target sequence in the same orientation

**fastx-fetch.pl** Retrieving sequences from a FASTQ/FASTA file given a a list of identifiers (possibly as a text file)

**fastx-length.pl** Generating length information and aggregate statistics for a FASTQ/FASTA file

**length_plot.r** Generating "digital electrophoresis" image and read density plots given a file containing length information

**porejuicer.py** Extracting raw data and called FASTQ files from FAST5 files

A rough shell command script (including additional dead-end attempts at discovery & analysis) is provided for reproduction and/or extension of these findings to other investigations (see Supplementary File 6).

## Supplementary material

**Supplementary File 1:** Base calling summary from Albacore v0.7.5.

**Supplementary File 2:** Reference sequences used for the initial amplicon mapping.

**Supplementary File 3:** Reference sequences used for *Ifna* paralog mapping.

**Supplementary File 4:** R script and intermediate data files used for *Ifna*-family gene counting.

**Supplementary File 5:** R script and intermediate data files used for chimeric read filtering.

**Supplementary File 6:** Shell/process script for reproducing the data analysis.
