# Supplementary material for: Investigation of chimeric reads using the MinION
Source: F1000Res. 2017 Aug 16;6:631. Originally published 2017 May 5. [Version 2] doi: 10.12688/f1000research.11547.2 (PMC5600009; doi:10.12688/f1000research.11547.2)
Supplement: Peer review history

# Peer review: Referee response for version 2

**DOI:** 10.5256/f1000research.13356.r25051

**Published:** 2017-09-13

**Authors:** Winston Timp

**Affiliations:** 1 Department of Biomedical Engineering, Johns Hopkins University, Baltimore, MD, USA

The question regarding the low number of 2D sequences was addressed by the authors as the effect of different versions of basecalling algorithm and sequencing chemistry. The authors further explained the differences in ligation conditions, concluding that the short ligation is better at preventing chimeric reads than long ligation. Based on these observations I am satisfied with the report.

I have read this submission. I believe that I have an appropriate level of expertise to confirm that it is of an acceptable scientific standard.

# Peer review: Referee response for version 1

**DOI:** 10.5256/f1000research.12473.r23252

**Published:** 2017-06-19

**Authors:** Winston Timp

**Affiliations:** 1 Department of Biomedical Engineering, Johns Hopkins University, Baltimore, MD, USA

In this manuscript, the authors discuss the detection and potential sources of chimeric reads from minION (nanopore) sequencing. Though the manuscript has some interesting analyses and ideas, I have some problems with the results that should be addressed, specifically points 1, 3, 4, 5 and 7:

1. Though 2D libraries were prepared - only *0.3%* of the reads were actually 2D, the vast majority were 1D. This is in my view quite surprising - though with 2D libraries I have seen plenty of 1D reads mixed in, this level of 2D/1D for a 2D prep suggests something strange upstream of sequencing is occurring. However, the authors decline to address it, “The reasons behind this basecall failure were not investigated”.  I think this must be addressed more carefully to understand the results.
2. I feel the low % of 2D reads is important because it may play into the source of the chimeras - if the 2D calling is failing due to heterogenous DNA strands - i.e. hybridization of an IFN to an Actb for example, then end polishing and adapting would lead to a 2D read where the two strands don’t match, hence called as 1D.
3. The authors suggest that amplicon sequencing is more susceptible to chimeras because “the same sequencing will appear in increased abundance” - I’m not clear on why that makes chimeras more frequent, just that it makes them more likely to be easily detectable.  
4. The authors discuss “multiple hairpin adapters being ligated in the same sequence”. I don’t understand how the authors think this is possible? There are only two free ends of DNA, and if there are hairpins on both, the DNA will not be able to enter the pore. Instead I suggest it could be the proposed “in silico” chimerism the authors later discuss.
5. PCR Chimeras are not unknown in the literature - having been described, for example, in Sanger and 454 here (PMID: 21212162, 20833233). The authors’ assumption that the chimerism is occurring downstream of PCR needs to be demonstrated - Figure 3 suggests that the length of the chimeric is not outside the range of either Illumina or Sanger sequencing, so could be easily validated with these technologies.
6. But - given the multiple ligation steps of this protocol, it seems likely that the dA-tailing failing some fraction of the time could results in blunt-end ligation and chimeric reads.
7. How does enrichment look comparing the overnight to quick ligation for the different categories of chimerism detailed? The only results given are overall chimerism.
8. The authors only tried overnight ligation/quick ligation for the last ligation step, but not for the barcode ligation step. I also wonder if a PCR-barcode may have given better results - the multiple ligations may have led to a higher rate of chimeras, as the end-polishing likely had some fraction of blunt-ended amplicons.
9. Another possible point was the the authors may have not added enough (relative) adaptors - the relative high concentration of template allowed self-ligations to be more frequent. Adaptor dimers are probably easier to eliminate in this case than chimeras.

I have read this submission. I believe that I have an appropriate level of expertise to confirm that it is of an acceptable scientific standard, however I have significant reservations, as outlined above.

# Response

**Published:** 2017-06-20

**Authors:** David Eccles

**Affiliations:** Malaghan Institute of Medical Research, New Zealand

Thanks for you comments. We will be incorporating your suggested changes into a revised manuscript. To make sure we've got the right idea about your suggestions, here are my initial impressions:

1. I understand what happened now after talking to Forrest Brennen at the London Calling conference this year. 2D reads were not called because the older hairpin was used. Even though we used a newer 2D kit, the barcode kit had the old hairpin sequence in it (which wasn't detected by the Albacore caller used at the time). This mis-call has either been fixed now, or will be fixed soon -- I'll re-call the reads with the most recent Albacore and if no improvement will talk to ONT about the correct software tweaks to fix it. In any case, plenty of hairpin sequences were detected in the linear/1D base calls.
2. We initially thought that the failure of 2D was due to chimeric reads, because it seemed to be occurring at the 2D alignment step. However, reads that were very obviously not chimeric were still failing the 2D calling. There is a chance that a properly called chimeric read will fail the 2D alignment step, but I expect it will still have a called template + complement sequence. I don't recall seeing many situations where the chimerism had happened on a single strand; it was mostly double-stranded fragments that had joined together.
3. My theory on why amplicon sequencing is more susceptible to chimeras is that it encourages the formation of base pairing structures (e.g. quadruplexes) that bring the ends of similar sequences closer to each other. I don't know how deep we should go into this; it's a hypothesis about why they could be in higher abundance for our specific experiment, but we haven't tested whether or not amplicon sequencing runs have a higher rate of chimeric reads.
4. Multiple hairpin adapters do make sense; David Stoddart (ONT sample prep guru) was with me when I was doing some "napkin drawings" of the structure that was formed by a 3-hairpin sequence, and helped correct a bit of the structure. We should add that into the paper (I think he kept the drawing, but I can make another one).
5. PCR chimeras may exist in our results, but appear to be of low abundance according to the electrophoresis plot, and I've tried to analyse the results in such a way that PCR chimeras would be excluded. Our experimental design was such that barcoding (and mixing of separate amplicons) happened after the PCR was done, so the results should be at worst an underestimate of chimerism, and PCR chimeras should be observable in the data.
6. Yes; dA-tailing failure seems to be a likely explanation. Due to the physics of chemical reactions, there are going to be some that don't work. However, it is a little bit curious that the overnight ligation produced more chimeric reads. If the chimerism were due solely to dA-tailing failure, then increased abundance for overnight ligation doesn't make sense.
7. Different categories of chimerism are outlined in Table 1, and the four categories are broken down into overnight/quick in figure 5. I've included a full text description of each *read* in the supplementary information, but we felt that it was overly complex to include all those details in a graph.
8. The overnight/quick difference was unexpected (particularly in the direction that it happened). While ONT have discontinued their 2D hairpin kit, we would still be able to carry out a subsequent investigation in the future to look at overnight vs quick ligation during barcoding.
9. We tried to add the recommended amount of adapters to the samples, and the called results suggest that adapter dimers were minimal. Those situations are very easy to pull out at the analysis stage, because there are no amplicon sequences between adapters.

# Peer review: Referee response for version 1

**DOI:** 10.5256/f1000research.12473.r22553

**Published:** 2017-05-17

**Authors:** Keith E. Robison

**Affiliations:** 1 Warp Drive Bio, Cambridge, MA, USA

The authors have provided a useful report on technical aspects of the emerging Oxford Nanopore MinION DNA sequencing technology.  As noted in the paper, the library preparation methods scrutinized here are commonly found in multiple advanced DNA sequencing technologies, and so lessons learned in this work are likely applicable elsewhere.

 In the methods section, the authors report injected "dead infectious" worms, but not how the worms were killed.

 It would be greatly preferable for all of the input DNA amounts to library preparations to be given in both mass and fmol.  While it is common to report masses of DNA, the ligations really are dependent on the availability of DNA ends. 

 The point Figure 8 is trying to convey would be greatly enhanced by adding a zoom of the region around 8s in the plot in which the temporal sequence barcodeNB11-open pore--stall-barcode NB06 is seen.  Zooms of other transitions should be considered as well.

I have read this submission. I believe that I have an appropriate level of expertise to confirm that it is of an acceptable scientific standard.
